# Why most patients do not exhibit obstructive sleep apnea after mandibular setback surgery?

**DOI:** 10.1186/s40902-020-00250-x

**Published:** 2020-03-17

**Authors:** Jin-Wook Kim, Tae-Geon Kwon

**Affiliations:** Department of Oral and Maxillofacial Surgery, School of Dentistry, Kyungpook National University, 2177 Dalgubeol-daero, Jung-gu, Daegu, 41940 Republic of Korea

**Keywords:** Mandibular setback, Obstructive sleep apnea, Airway, Prognathism

## Abstract

Maxillomandibular advancement (MMA) is effective for the treatment of obstructive sleep apnea (OSA). In previous studies, the airway was increased in the anteroposterior and transverse dimensions after MMA. However, the effect of the opposite of mandibular movement (mandibular setback) on the airway is still controversial. Mandibular setback surgery has been suggested to be one of the risk factors in the development of sleep apnea. Previous studies have found that mandibular setback surgery could reduce the total airway volume and posterior airway space significantly in both the one-jaw and two-jaw surgery groups. However, a direct cause-and-effect relationship between the mandibular setback and development of sleep apnea has not been clearly established. Moreover, there are only a few reported cases of postoperative OSA development after mandibular setback surgery.

These findings may be attributed to a fundamental difference in demographic variables such as age, sex, and body mass index (BMI) between patients with mandibular prognathism and patients with OSA. Another possibility is that the site of obstruction or pattern of obstruction may be different between the awake and sleep status in patients with OSA and mandibular prognathism. In a case-controlled study, information including the BMI and other presurgical conditions potentially related to OSA should be considered when evaluating the airway. In conclusion, the preoperative evaluation and management of co-morbid conditions would be essential for the prevention of OSA after mandibular setback surgery despite its low incidence.

## Background

For patients with prognathism or skeletal class III malocclusion, mandibular setback surgery is frequently performed to improve esthetic and functional problems. Although mandibular setback surgery can greatly improve the patients’ chief complaints, several recent reports and a systematic review on airway changes after mandibular setback surgery have shown significant changes in the pharyngeal airway volume or posterior pharyngeal airway space (PAS) [1, 2]. However, it is controversial that mandibular setback surgery can induce postoperative obstructive sleep apnea (OSA). In a previous study, two patients with a large mandibular setback (13.7 mm and 12.6 mm) developed mild OSA 6 months after mandibular setback surgery [3]. In addition, a recent study reported that among 12 patients with a mandibular setback exceeding 10 mm, 4 patients developed postoperative OSA, and the apnea-hypopnea index (AHI) was slightly increased significantly after the surgery [4]. Another study found that although subjective symptoms were not exacerbated, objective sleep quality was decreased after mandibular setback surgery [5]. When mandibular setback was performed with maxillary advancement or posterior impaction, the pharyngeal airway volume significantly decreased. However, it did not significantly affect AHI values or induce OSA [6]. Furthermore, two systematic reviews indicated that there was no clear evidence to confirm whether two-jaw surgery or isolated mandibular surgery may be a causative factor of OSA development [1, 7]. Currently, a direct cause-and-effect relationship between the mandibular setback and development of sleep apnea has not been clearly established.

Positive airway pressure therapy is primarily recommended for patients with severe OSA. However, when patients with severe OSA cannot tolerate the therapy, maxillomandibular advancement (MMA) is recommended as a surgical option to increase the airway space [8]. A meta-analysis of the efficacy of MMA for treating OSA has been conducted using the results from 45 studies involving 455 patients [9] and clearly showed MMA can improve airway patency. In previous assessments of three-dimensional (3D) airway changes after MMA, the total airway volume was significantly increased [10–12]. The increase in the transverse airway was even greater than that in the anteroposterior airway [13].

Based on the results of previous studies involving mandibular setback, in the reverse direction of mandibular advancement, it can be assumed that there would be many OSA patients or a risk of OSA development after mandibular setback surgery. Various studies have indicated the possibility of sleep-disordered breathing after mandibular setback surgery. However, there are only a few reported cases of postoperative OSA development. The purpose of this review is to investigate various factors related to postoperative airway changes after mandibular setback surgery and answer the key question: why most patients do not exhibit OSA after a significant amount of mandibular setback?

## Review

Orthognathic surgery that moves maxillomandibular structures could affect skeletal structures and related soft tissues including the soft palate, tongue, and epiglottis. Nasopharyngeal and oropharyngeal airways are significantly affected by the direction and amount of maxilla and mandibular movements [1, 11, 12]. Advancement surgeries such as MMA could significantly improve the oropharyngeal airway; however, the posterior movement of the mandible usually narrows the airway [1, 2, 8, 9]. In this review, the difference in the results of MMA and mandibular setback was compared, and potential factors related to the development of OSA after mandibular setback surgery were reviewed.

### Significant airway reduction after mandibular setback surgery

Various studies on mandibular setback surgery have reported that the airway volume was significantly decreased. Based on a review of previous literature on the pharyngeal airway volume, the total pharyngeal airway volume (TPV) was decreased to 75.2 ~ 80.9% after surgery and recovered to 70.7 ~ 87.7% over 1 year after surgery compared with the presurgical volume [14–19] (Table 1). It was also reported that two-jaw surgery with mandibular setback and maxillary advancement or with maxillary posterior impaction could induce significant airway volume reduction after surgery [4, 6, 14–16, 18, 20–24]. The TPV was decreased to 82.0 ~ 96.8% in the short term after surgery and recovered to 84.5 ~ 98.0% around 6 months after surgery (Table 2). However, the degree of airway reduction was smaller than that in mandibular setback surgery alone [6, 14, 16, 25, 26]. Immediately after surgery, airway narrowing was more severe because of soft tissue edema. Although airway narrowing was improved after surgery, the pharyngeal airway volume remained lower compared with the preoperative airway volume. The comparative studies and meta-analysis of mandibular setback surgery have shown that maxillary advancement or maxillary posterior impaction would be able to attenuate the narrowing of the airway [2, 27].

**Table 1:** Changes in the total pharyngeal airway volume after isolated mandibular setback surgery for mandibular prognathism

Ref.	*n*	Age (years, mean ± SD, range)	BMI	Surgery	Follow-up	Total pharyngeal airway volume (cm^3^)			
						T0^a^	T1	T2	T3
Park JW (2010) [17]	12	25.5		MnS	Pre and post 6 mo	17.6 (100.0%)		16.1 (91.3%)	
Hong JS (2011) [15]	12	23.2 ± 3.6		MnS (no skeletal movement information)	Pre and post 2 mo	8.5 (100.0%)	6.9 (80.9%)		
Park SB (2012) [18]	20	Total 23.0 ± 3.0 (19–29)		MnS (setback 7.9 ± 3.6 mm)	Pre and post 5 mo and post 17 mo	36.6 (100.0%)		32.4 (88.5%)	32.1 (87.7%)
Hatab (2015) [14]	9	Total 21.8 ± 3.4 (18–30)		MnS (no skeletal movement information)	Pre and post 3 mo	30 (100.0%)	22.5 (75.2%)		
Shah (2016) [19]	29	23.7 ± 6.3 (18–52)		MnS (setback 7.7 mm)	Pre and post 6 mo and 1 year	35.5 (100.0%)		24.4 (68.7%)	25.1 (70.7%)
Lee ST (2019) [16]	25	23.0 ± 4.4	22.4 ± 3.5	MnS (setback 9.1 ± 2.6 mm)	Pre and post 1 mo and 1 year	26 (100.0%)	19.6 (75.4%)		22.1 (85.0%)
Total						**(100.0%)**	**(77.1%)**	**(82.9%)**	**(81.1%)**

*MnS* mandibular setback surgery (amount of setback), *Pre* preoperative, *Post* postoperative, *mo*, months

^a^The preoperative state (T0) was set as 100%. T0, preoperative; T1, postoperative 1–4 months; T2, postoperative 5–6 months; T3, postoperative 1 year. The total age indicates that the study did not define the value for individual study groups

**Table 2:** Changes in in the total pharyngeal airway volume after two-jaw surgery for mandibular prognathism

Ref.	*n*	Age (years, mean ± SD, range)	BMI	Surgery	Follow-up	Total pharyngeal airway volume (cm^3^)			
						T0^a^	T1	T2	T3
Hong JS (2011) [15]	9	22.2 ± 4.8		MnS + MxA(?) (no skeletal movement information)	Pre and post 2 mo	8.2 (100.0%)	7.1 (86.8%)		
Lee Y (2012) [23]	21	22.7 (18.1–33.4)	20.8 ± 2.6 (17.6–26.1)	MnS (setback 9.2 ± 4.6 mm), Mx pst impaction (5.3 ± 2.6 mm)	Pre and post 3 mo and 6 mo	25.1 (100.0%)	24.3 (96.8%)	24.6 (98.0%)	
Park SB (2012) [18]	16	Total 23.0 ± 3.0 (19–29)		MnS (setback 4.2 ± 1.7 mm), MxA (adv 7.2 ± 3.4 mm)	Pre and post 5 mo and 17 mo	38.2 (100.0%)		33.9 (88.7%)	36.2 (94.8%)
Li YM (2014) [24]	29	23.6 (18–35)	< 30	MnS (setback 5.8 ± 1.7 mm), MxA (adv 3.5 ± 0.8 mm)	Pre and post 6 mo	28.51 (100.0%)		26.54 (93.1%)	
Kim MA (2014) [22]	25	23.7 ± 4.3		MnS (setback 8.8 ± 5.5 mm), Mx pst impaction (3.4 ± 2.2 mm)		44 (100.0%)		42.3 (96.1%)	
Hsieh (2015) [20]	32	24.0 ± 3.9 (18–32)	20.1 ± 2.5	MnS (setback 7.0 mm), Mx pst impaction (3.4 mm)	Pre and post 6 mo	23.1 (100.0%)		20.4 (88.3%)	
Hatab (2015) [14]	11	Total 21.8 ± 3.4 (18–30)		MnS + MxA (no skeletal movement information)	Pre and post 3 mo	30.3 (100.0%)	27.27 (90.0%)		
Kim HS (2016) [21]	38	23.8 ± 5.9 (17–44)	21.1 ± 2.7	MnS (setback 6.2 ± 3.1 mm), Mx post impaction	Pre and post 3 mo and 6 mo	23.4 (100.0%)	20.08 (85.8%)	20.09 (85.9%)	
Jang SI (2018) [6]	13	23.9 ± 5.2	24.9 ± 2.5	MnS (setback 10.2 ± 3.3 mm), Mx pst impaction (3.9 ± 1.7 mm)	Pre and post 7 mo	15.95 (100.0%)		13.48 (84.5%)	
Lee ST (2019) [16]	23	23.3 ± 4.2	24.5 ± 4.5	MnS (setback 9.9 ± 4.0 mm), Mx pst impaction (3.4 ± 1.7 mm)	Pre and post 1 mo and 1 year	30.5 (100.0%)	25 (82.0%)		26.7 (87.5%)
Yang HJ (2020) [4]	12	21.8 ± 2.9	21.1 (17.8–25.2)	MnS (setback 11.8 mm, 9.6 ~ 14.3 mm) Mx pst impaction (3.82 mm)	Pre and post 4–6 mo	23 (100.0%)		21.1 (91.7%)	
Total						**(100.0%)**	**(88.3%)**	**(90.8%)**	**(91.2%)**

*MnS* mandibular setback surgery (amount of setback), *MxA* maxillary advancement (adv), *Mx pst impaction* maxillary posterior impaction, *Pre* preoperative, *Post* postoperative, *mo* months.

^a^The preoperative state (T0) was set as 100%. T0, preoperative; T1, postoperative 1–4 months; T2, postoperative 5–6 months; T3, postoperative 1 year. The total age indicates that the study did not define the value for individual study groups

The AHI is defined as the total number of apneas and hypopneas per hour of sleep by polysomnography. There have been several reports on the AHI before or after mandibular setback surgery [3–6, 26, 28, 29]. The categories for OSA can be defined as follows: normal, AHI < 5; mild sleep apnea, 5 ≤ AHI < 15; moderate sleep apnea, 15 ≤ AHI < 30; severe sleep apnea, AHI ≥ 30 [30]. The average AHI of patients was reported to range from 0.4 to 3.1 before surgery and 1.1 to 4.75 after surgery. The estimated total average of the preoperative and postoperative AHI was 1.9 ± 0.8 and 2.3 ± 1.1, respectively (Table 3). Based on the OSA categories, most studies demonstrated that the average AHI after mandibular setback surgery with or without maxillary advancement remained normal even though there was a slight increase in the AHI value after surgery. According to World Health Organization definitions, BMI ≥ 30 kg/m^2^ can be defined as obesity, and BMI 25–29.9 kg/m^2^ can be defined as overweight [31]. Since the most of the studies on airway changes after mandibular setback surgery frequently included non-obese or non-overweight (BMI < 25) and non-OSA patients before surgery, it can be hypothesized that there may be a lower risk of OSA development among these patients [3, 6, 16, 20, 21, 23, 26, 28, 29, 32] (Table 3).

**Table 3:** Age, body mass index (BMI), and apnea-hypopnea index (AHI) of the studies investigating pharyngeal airway changes in mandibular setback surgery with or without maxillary surgery

	Age (years)		BMI before surgery (kg/m^2^)		AHI (before surgery)		AHI (after surgery)	
	Average	± SD or range	Average	± SD or range	Average	± SD or range	Average	± SD or range
Hasebe (2011) [3], 1 + 2 jaw	22		21.3	17.2–33.7	1-jaw 2.2; 2-jaw 2.9	1-jaw 3.3; 2-jaw 3.3	1-jaw 2.7; 2-jaw 2.1	1-jaw 3.4; 2-jaw 1.7
Gokce (2012) [28], 2 jaw	20.9	± 0.8	22.3	± 4.2	2.1	2.74	1.4	1.75
Lee Y (2012) [23], 2 jaw	22.7	18.1–33.4	20.8	± 2.6				
Kobayashi (2013) [32], 1 + 2 jaw	24	16–38	21.4	16.1–30.9				
Uesugi (2014) [26], 1 + 2 jaw	23	16–54	21.1	15–34.4	1-jaw 3.1; 2-jaw 1.9	1-jaw 3.2; 2-jaw 1.7	1-jaw 3.4; 2-jaw 2.2	1-jaw 4.1; 2-jaw 2.1
Hsieh (2015) [20], 2 jaw	24	± 3.9	20.1	± 2.5				
Kim HS (2016) [21], 2 jaw	23.8	± 5.9	21.1	± 2.7				
Jang SI (2018) [6], 2 jaw	23.9	± 5.2	24.9	± 2.5	2.24	1.24	4.75	5.91
Lee ST (2019) [16], 1 + 2 jaw	23.3	± 4.2	24.5	± 4.5				
On SW (2019) [5], 1 + 2 jaw	21.86	± 4.55	22.65	15.3–33.3	1.15	0–12.4	1.1	0–28.7
Schilbred Eriksen (2019) [29], 1 jaw	23.2	18.2–33.4	24.2	20.1–32.1	1.3	0–2.5	1.8	0.3–3.3
Yang HJ (2020) [4], 2 jaw	21.8	± 2.9	21.1	17.8–25.2	0.4	0–2.9	1.2	0–8.2
Total	**22.8 ± 1**		**22.1 ± 1.5**		**1.9 ± 0.8**		**2.3 ± 1.1**	

A narrowing airway can eventually lead to the development of breathing problems and OSA after surgery, as indicated by several studies [3, 15, 28, 33]. Postoperative OSA development after mandibular setback surgery has been reported in several studies [3, 4, 26]. Table 4 summarizes the information for seven reported cases of OSA after mandibular setback surgery. The overall amount of mandibular setback was large in these patients (9.61–14.26 mm) compared with patients in the previous studies, which investigated airway changes after mandibular setback surgery shown in (Table 1). One patient who developed postoperative OSA already had a high BMI and AHI before surgery [26]. Interestingly, even for an underweight patient with a normal AHI (BMI of 18.9, preoperative AHI of 0.2), the AHI was increased to 7 [4].

**Table 4:** Reported obstructive sleep apnea after mandibular setback surgery

	Amount of mandibular setback (mm)	Age	Sex	BMI before surgery (kg/m^2^)	AHI (before surgery)	AHI (after surgery)	OSA category after surgery
Hasebe (2011) [3] (*n* = 2/22)^a^	13.7 (at Pog)	22	M	20.6 healthy	4.4	12.1	Mild sleep apnea
	12.6 (at Pog)	18	F	21.3 healthy	2.1	5.4	Mild sleep apnea
Uesugi (2014) [26] (*n* = 1/40)^a^	10.1 (at Pog)	54	M	34.4 obesity	14.9	19	Severe sleep apnea
Yang HJ (2020) [4] (*n* = 4/12)^a^	12.88 (at B)	23	F	24.2 healthy	2	8.2	Mild sleep apnea
	9.61 (at B)	22	M	25.2 overweight	1.3	6.3	Mild sleep apnea
	14.26 (at B)	22	M	18.9 healthy	0.2	7	Mild sleep apnea
	11.56 (at B)	22	M	21.5 healthy	2.9	5.2	Mild sleep apnea
Total	**12.1 ± 1.8**	**26.1 ± 12.4**	**M = 5; F = 2**	**23.7 ± 5.2**	**4.0 ± 5.0**	**9.0 ± 5.0**	

^a^Incidence of OSA after surgery. BMI category: obesity, ≥ 30; overweight, 25–29.9; healthy weight, 18.5–24.9; underweight, < 18.5 [31]. OSA category: normal, AHI < 5; mild sleep apnea, 5 ≤ AHI < 15; moderate sleep apnea, 15 ≤ AHI < 30; severe sleep apnea, AHI ≥ 30 [30]

However, a recent review of previously published systematic reviews suggested that although the development of postsurgical OSA has been reported, there is no clear evidence that confirms a direct cause-and-effect relationship between mandibular setback surgery and OSA development [27]. For patients with severe mandibular prognathism or patients with potential for OSA development, two-jaw surgery should be strongly considered [34]. In addition, a study reported that although there are no subjective symptoms after mandibular setback with or without maxillary surgery, objective sleep quality determined by polysomnography may be decreased [5]. However, a short-term observation period (3 months) needs to be considered in this previous study.

### Improvement of airway patency by MMA

MMA has been frequently reported to be an effective method to treat or reduce OSA severity [9, 11, 35] and has shown a high treatment success rate (> 85%) [36]. Since mandibular setback surgery is in the reverse direction of mandibular advancement, an objective comparison of previous findings for the two different procedures is needed.

According to a systematic review and meta-analysis based on 45 studies, the preoperative AHI (57.2 ± 25.4) was significantly decreased to 9.5 ± 10.4, and the preoperative BMI of OSA patients was 33.8 ± 9.7 with an average age of 45.3 ± 10.0 years [9]. In the current review, the average age of patients who underwent MMA ranged from 33 to 53.8 years (average 45.7 ± 5.4 years) based on previous studies [12, 13, 33, 37–47] (Table 5). Most of the studies included overweight patients (BMI > 25), and the average preoperative AHI was 51.1 ± 12.6. The average AHI after MMA ranged from 4.8 to 29.4 (12.3 ± 7.5). Interestingly, a comparison of the results of Tables 3 and 5 revealed that although the AHI was greatly improved after MMA, the value was still higher than the average postoperative AHI after mandibular setback surgery (range, 1.1–4.75; average, 2.3 ± 1.1; Table 3). The improved (decreased) AHI after MMA for OSA was still higher than the increased AHI after mandibular setback. A comparison of the airway dimension revealed that the improved PAS (from 5.5 ± 2.8 mm to 11.5 ± 3.8 mm) after MMA for OSA [9] was still similar or narrower than the decreased PAS after mandibular setback surgery (preoperative PAS, 9.6–25.1 mm; postoperative PAS, 9.6–20.3 mm) [1]. Most of the patients who underwent mandibular setback surgery were young and not obese on average compared with patients who underwent MMA. Notably, the range of the patients’ age, BMI, and perioperative AHI was different between the two groups (mandibular setback vs. MMA).

**Table 5:** Age, body mass index (BMI), and apnea-hypopnea index (AHI) of the studies investigating pharyngeal airway changes in maxillomandibular advancement (MMA)

	Age (years)		BMI before surgery (kg/m^2^)		AHI (before surgery)		AHI (after surgery)	
	Average	± SD or (range)	Average	± SD or (range)	Average	± SD or (range)	Average	± SD or (range)
Conradt (1997) [37]	44	± 12	28.3	± 3.4	51.4	± 16.9	8.5	± 9.4
Li (2000) [38]	45.6	± 20.7	31.4	± 6.7	71.2	± 27.0	7.6	± 5.1
Fairburn (2007) [33]	47.6	± 10	32.24	4.7	69.2	± 35.8	18.6	± 6.3
Jones (2010) [39]			33.9	± 8.5	61.41	± 19.6	29.4	± 19.4
Dekeister (2006) [40]	48	± 7	28	3.4	45.4	± 18.1 (30–88)	8.3	± 6.8 (1–32)
Jaspers (2013) [41]	53.8	± 9.1			36.2	± 23.8 (16–81)	11.3	± 16.1 (1–43)
Ronchi (2013) [42]	42.3	± 9.5			58.7	± 16	8.1	± 7.8
Bianchi (2014) [12]	45	± 14			56.8	± 16.6	12.3	± 5.5
Schendel (2014) [13]	46.4	± 9.7	28.6		42.9	± 21.2	5.2	± 8.3
Hsieh (2014) [43]	33	± 7.9	22	3.3	35.7	± 18.0	4.8	± 4.4
Boyd (2015) [44]	48.4	± 8.1	29.1	4.1	50.4	± 19.7 (17–87.6)	8	± 10.7 (0.2–41.7)
Vigneron (2016) [45]	40.7	± 12.6	24.6	4	56.7	± 23.9	25.5	± 20.6
Veys (2017) [46]	44.7	± 9.5			27.7	± 14.7	8.5	± 10
de Ruiter (2017) [47]	54	47–61	29	27–33	52.1	± 10	16	± 10
Total	**45.7 ± 5.4**		**28.7 ± 3.5**		**51.1 ± 12.6**		**12.3 ± 7.5**	

### Factors related to rare OSA development after mandibular setback

Three factors can explain the sporadic incidence of postoperative OSA after mandibular setback surgery despite the presence of data showing airway narrowing after surgery: age and gender, BMI, and study design.

#### Age and gender

Age can influence sleep by affecting pharyngeal collapsibility during sleep. Aged patients have been reported to show increased pharyngeal collapsibility during sleep [48]. As the age increases, the BMI usually increases with changes in the muscle tone, connective tissue flaccidity, and adipose tissue distribution [49]. However, younger age and female gender are less likely to be affected by airway collapse [50]. Interestingly, there is a fundamental difference between males and females in terms of pharyngeal airway anatomy or tissue characteristics. Males are more susceptible than females to load-induced hypoventilation because of the increased airway collapse [51]. There is a vicious cycle between poor sleep and low testosterone levels. A low testosterone level results in a higher OSA risk [52]. Estrogen protects against OSA by exerting anti-depressant and sleep effects. A reduced estrogen level could affect the level of serotonin, which controls the tongue and palate muscle tone [53]. In women who are pregnant or with menopause, the OSA risk is higher [54]

As shown in Table 5, patients who underwent MMA are usually older and male compared with patients who underwent mandibular setback surgery. The lack of postoperative OSA after mandibular setback surgery may be attributed to a fundamental difference in demographic variables between patients with mandibular prognathism and patients with OSA. Therefore, if mandibular setback surgery is needed for aged, male patients with a high BMI, it is important to inform the patients of the potential risk of postoperative OSA even though it is very rare.

#### BMI

OSA is known to affect multiple body systems. The prevalence of OSA is increased in obese subjects. Obesity is a predisposition to OSA [55, 56]. An excessive body weight results in structural and functional changes in the upper airway and changes the balance between ventilation and perfusion. OSA patients usually have a more narrow and collapsible pharynx and show a larger cross-sectional area of adipose tissue adjacent to the pharyngeal airway [57]. According to autopsy data, lingual fat is distributed mainly at the posterior tongue (30%) rather than the anterior tongue (11%) or other somatic muscles (3%). Furthermore, lingual fat is significantly correlated with the BMI [58]. The tongue volume is increased in patients with OSA. Fat deposition at the base of the tongue has been found to be significantly increased in patients with OSA compared with control patients without OSA. This finding may also explain the significant effect of obesity on OSA [59].

**Fig. 1:**
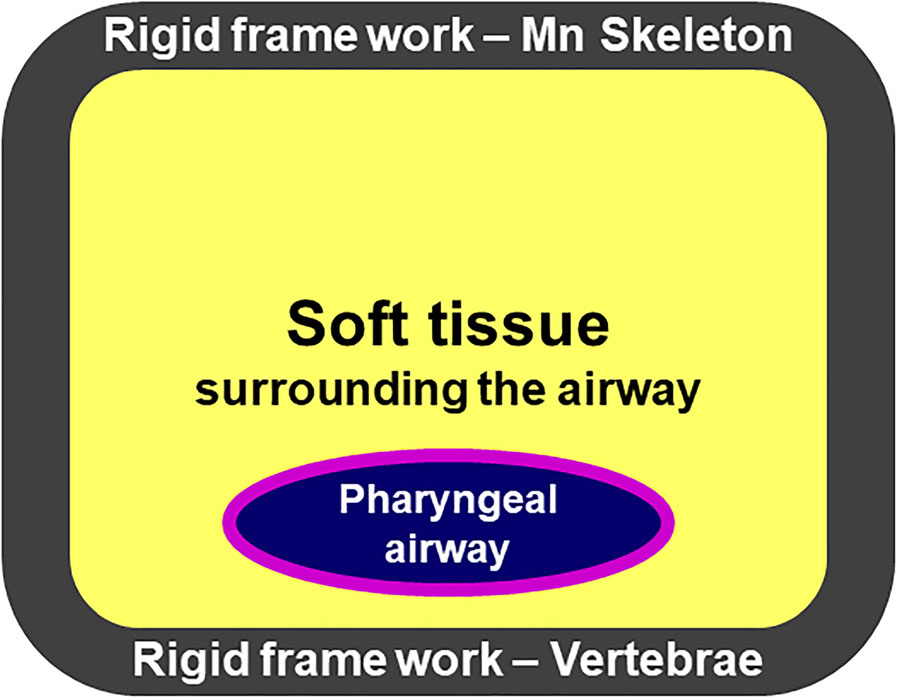
Relationship between obesity and OSA. The mandibular skeleton or vertebrae can serve as a rigid framework. Increased fat accumulation at the pharyngeal airway can increase tissue pressure surrounding the pharyngeal airway. The lung volume may be decreased by excessive visceral fat deposition in the chest cavity between the vertebrae and ribs

The relationship between OSA and obesity can be explained by the increased collapsibility of the airway due to substantial fat accumulation in two areas: the pharyngeal airway and lungs (Figure 1). First, the pharyngeal airway may be decreased due to an excess of soft tissue content relative to the existing size of the mandibular skeleton. This can increase tissue pressure surrounding the pharyngeal airway. Second, the lung volume may be decreased by excessive visceral fat deposition in a given chest volume between the vertebrae and rib cage. These can reduce tracheal traction forces and tension in the pharyngeal wall [60]. Therefore, an efficient strategy for the treatment of OSA would be to increase the framework size or reduce the soft tissue volume. On the other hand, the development of postoperative OSA after mandibular setback surgery could be related to the reduction of both the mandibular skeletal framework size and the soft tissue surrounding the pharyngeal airway. It can be hypothesized that the collapsibility of the pharyngeal airway is different between patients with OSA and patients with mandibular prognathism. In addition, a considerable BMI difference between patients with mandibular prognathism and patients with OSA can explain the rare occurrence of postoperative OSA after mandibular setback surgery.

#### Study design

A number of studies investigating airway changes after mandibular setback surgery have fundamental problems in their design including the lack of a normal control group. They mostly compared one-jaw and two-jaw surgery without data for the normal control. The lack of a matched control group in terms of gender, age, or BMI results in uncertainty whether the airway volume reduction after mandibular setback indicates a real risk of OSA. Moreover, most previous studies did not examine the preoperative BMI or sleep disorders; thus, it is difficult to clarify whether postoperative OSA is caused by the aggravation of preexisting conditions or primarily by the surgery itself. To the best of our knowledge, only one study has compared the preoperative and postoperative pharyngeal airway in cases of mandibular setback surgery with maxillary surgery using age- and sex-matched control subjects [20]. According to this previous study, the TPV was significantly reduced from 23.1 cm^3^ to 20.4 cm^3^ after surgery. The reduced TPV after surgery was not lower in the experimental group compared with the age-, sex-, and BMI-matched normal control group (17.6 cm^3^). In a systematic review by Canellas et al., airway volume reduction in mandibular setback surgery and a decreased minimum cross-sectional area were not accompanied by signs and symptoms of OSA [1]. The association can be better investigated by a well-designed case-control clinical study with a larger number of normal subjects.

Recently, it has been revealed that obstruction sites may not be visible on conventional radiographic evaluation or awake clinical examination. Therefore, drug-induced sleep endoscopy is widely used for a reliable assessment of the level and degree of upper airway obstruction [61]. The site of obstruction or pattern of obstruction may be different between the awake and sleep status in patients with OSA and mandibular prognathism. Therefore, this should be considered in the detection and analysis of potential postoperative OSA after mandibular setback.

## Conclusion

Mandibular setback surgery may be a risk factor for sleep-disordered breathing considering the evidence of decreased pharyngeal airway volume after surgery. However, there are only a few reported cases of postoperative OSA after mandibular setback surgery. It is possible that risk factors for OSA development, such as older age, high BMI, and male gender, are not prevalent in the study population. The BMI and presurgical conditions should be considered in the evaluation of the airway. Therefore, the preoperative evaluation and management of co-morbid conditions would be essential for the prevention of OSA after mandibular setback surgery. Patients having high-risk factors for OSA development need to be aware of potential postoperative OSA after mandibular setback surgery despite its low incidence.

## Data Availability

Not applicable. (Data sharing not applicable to this article as no datasets were generated or analyzed during the current study.)

## Abbreviations

AHI: Apnea-hypopnea index
BMI: Body mass index
MMA: Maxillomandibular advancement
OSA: Obstructive sleep apnea
PAS: Pharyngeal airway space
TPV: Total pharyngeal airway volume

## References

[1] Canellas JV, Barros HL, Medeiros PJ, Ritto FG (2016). Sleep-disordered breathing following mandibular setback: a systematic review of the literature. Sleep Breath.

[2] He J, Wang Y, Hu H, Liao Q, Zhang W, Xiang X, Fan X (2017). Impact on the upper airway space of different types of orthognathic surgery for the correction of skeletal class III malocclusion: a systematic review and meta-analysis. Int J Surg.

[3] Hasebe D, Kobayashi T, Hasegawa M, Iwamoto T, Kato K, Izumi N, Takata Y, Saito C (2011). Changes in oropharyngeal airway and respiratory function during sleep after orthognathic surgery in patients with mandibular prognathism. Int J Oral Maxillofac Surg.

[4] Yang HJ, Jung YE, Kwon IJ, Lee JY, Hwang SJ (2020). Airway changes and prevalence of obstructive sleep apnoea after bimaxillary orthognathic surgery with large mandibular setback. Int J Oral Maxillofac Surg.

[5] On SW, Kim HJ, Cho DH, Moon YR, Il Song S (2019). Silent changes in sleep quality following mandibular setback surgery in patients with skeletal class III malocclusion: a prospective study. Sci Rep.

[6] Jang SI, Ahn J, Paeng JY, Hong J (2018). Three-dimensional analysis of changes in airway space after bimaxillary orthognathic surgery with maxillomandibular setback and their association with obstructive sleep apnea. Maxillofac Plast Reconstr Surg.

[7] Fernandez-Ferrer L, Montiel-Company JM, Pinho T, Almerich-Silla JM, Bellot-Arcis C (2015). Effects of mandibular setback surgery on upper airway dimensions and their influence on obstructive sleep apnoea - a systematic review. J Craniomaxillofac Surg.

[8] Aurora RN, Casey KR, Kristo D, Auerbach S, Bista SR, Chowdhuri S, Karippot A, Lamm C, Ramar K, Zak R (2010). Practice parameters for the surgical modifications of the upper airway for obstructive sleep apnea in adults. Sleep.

[9] Zaghi S, Holty JE, Certal V, Abdullatif J, Guilleminault C, Powell NB, Riley RW, Camacho M (2016). Maxillomandibular advancement for treatment of obstructive sleep apnea: a meta-analysis. JAMA Otolaryngol Head Neck Surg.

[10] Hsieh YJ, Liao YF (2013). Effects of maxillomandibular advancement on the upper airway and surrounding structures in patients with obstructive sleep apnoea: a systematic review. Br J Oral Maxillofac Surg.

[11] Louro RS, Calasans-Maia JA, Mattos CT, Masterson D, Calasans-Maia MD, Maia LC (2018). Three-dimensional changes to the upper airway after maxillomandibular advancement with counterclockwise rotation: a systematic review and meta-analysis. Int J Oral Maxillofac Surg.

[12] Bianchi A, Betti E, Tarsitano A, Morselli-Labate AM, Lancellotti L, Marchetti C (2014). Volumetric three-dimensional computed tomographic evaluation of the upper airway in patients with obstructive sleep apnoea syndrome treated by maxillomandibular advancement. Br J Oral Maxillofac Surg.

[13] Schendel SA, Broujerdi JA, Jacobson RL (2014). Three-dimensional upper-airway changes with maxillomandibular advancement for obstructive sleep apnea treatment. Am J Orthod Dentofacial Orthop.

[14] Hatab NA, Konstantinovic VS, Mudrak JK (2015). Pharyngeal airway changes after mono- and bimaxillary surgery in skeletal class III patients: cone-beam computed tomography evaluation. J Craniomaxillofac Surg.

[15] Hong JS, Park YH, Kim YJ, Hong SM, Oh KM (2011). Three-dimensional changes in pharyngeal airway in skeletal class III patients undergoing orthognathic surgery. J Oral Maxillofac Surg.

[16] Lee ST, Park JH, Kwon TG (2019). Influence of mandibular setback surgery on three-dimensional pharyngeal airway changes. Int J Oral Maxillofac Surg.

[17] Park JW, Kim NK, Kim JW, Kim MJ, Chang YI (2010). Volumetric, planar, and linear analyses of pharyngeal airway change on computed tomography and cephalometry after mandibular setback surgery. Am J Orthod Dentofacial Orthop.

[18] Park SB, Kim YI, Son WS, Hwang DS, Cho BH (2012). Cone-beam computed tomography evaluation of short- and long-term airway change and stability after orthognathic surgery in patients with class III skeletal deformities: bimaxillary surgery and mandibular setback surgery. Int J Oral Maxillofac Surg.

[19] Shah DH, Kim KB, McQuilling MW, Movahed R, Shah AH, Kim YI (2016). Computational fluid dynamics for the assessment of upper airway changes in skeletal class III patients treated with mandibular setback surgery. Angle Orthod.

[20] Hsieh Yuh-Jia, Chen Yi-Chieh, Chen Yin-An, Liao Yu-Fang, Chen Yu-Ray (2015). Effect of Bimaxillary Rotational Setback Surgery on Upper Airway Structure in Skeletal Class III Deformities. Plastic and Reconstructive Surgery.

[21] Kim HS, Kim GT, Kim S, Lee JW, Kim EC, Kwon YD (2016). Three-dimensional evaluation of the pharyngeal airway using cone-beam computed tomography following bimaxillary orthognathic surgery in skeletal class III patients. Clin Oral Investig.

[22] Kim MA, Kim BR, Youn JK, Kim YJ, Park YH (2014). Head posture and pharyngeal airway volume changes after bimaxillary surgery for mandibular prognathism. J Craniomaxillofac Surg.

[23] Lee Y, Chun YS, Kang N, Kim M (2012). Volumetric changes in the upper airway after bimaxillary surgery for skeletal class III malocclusions: a case series study using 3-dimensional cone-beam computed tomography. J Oral Maxillofac Surg.

[24] Li YM, Liu JL, Zhao JL, Dai J, Wang L, Chen JW (2014). Morphological changes in the pharyngeal airway of female skeletal class III patients following bimaxillary surgery: a cone beam computed tomography evaluation. Int J Oral Maxillofac Surg.

[25] Yang Y, Yang K, Zhao Y (2018). Three-dimensional changes in the upper airway of skeletal class III patients after different orthognathic surgical procedures. J Oral Maxillofac Surg.

[26] Uesugi T, Kobayashi T, Hasebe D, Tanaka R, Ike M, Saito C (2014). Effects of orthognathic surgery on pharyngeal airway and respiratory function during sleep in patients with mandibular prognathism. Int J Oral Maxillofac Surg.

[27] Tan SK, Leung WK, Tang ATH, Zwahlen RA (2017). Effects of mandibular setback with or without maxillary advancement osteotomies on pharyngeal airways: an overview of systematic reviews. PLoS One.

[28] Gokce SM, Gorgulu S, Gokce HS, Bengi O, Sabuncuoglu F, Ozgen F, Bilgic H (2012). Changes in posterior airway space, pulmonary function and sleep quality, following bimaxillary orthognathic surgery. Int J Oral Maxillofac Surg.

[29] Schilbred Eriksen E, Gulati S, Moen K, Wisth PJ, Loes S (2019). Apnea-hypopnea index in healthy class III patients treated with intraoral vertical ramus osteotomy: a prospective cohort study. J Oral Maxillofac Surg.

[30] Ruehland WR, Rochford PD, O’Donoghue FJ, Pierce RJ, Singh P, Thornton AT (2009). The new AASM criteria for scoring hypopneas: impact on the apnea hypopnea index. Sleep.

[31] World Health Organization. Obesity and overweight. Fact sheet no 311 January 2015. (2016; Available from]) http://www.who.int/mediacentre/factsheets/fs311/en/

[32] Kobayashi T, Funayama A, Hasebe D, Kato Y, Yoshizawa M, Saito C (2013). Changes in overnight arterial oxygen saturation after mandibular setback. Br J Oral Maxillofac Surg.

[33] Fairburn SC, Waite PD, Vilos G, Harding SM, Bernreuter W, Cure J, Cherala S (2007). Three-dimensional changes in upper airways of patients with obstructive sleep apnea following maxillomandibular advancement. J Oral Maxillofac Surg.

[34] Al-Moraissi EA, Al-Magaleh SM, Iskandar RA, Al-Hendi EA (2015). Impact on the pharyngeal airway space of different orthognathic procedures for the prognathic mandible. Int J Oral Maxillofac Surg.

[35] Araujo PM, Osterne RLV, de Souza Carvalho ACG, Azevedo NO, Gondim RF, Goncalves Filho RT, Sant’Ana E, Nogueira RLM (2019). Pharyngeal airway space changes after maxillomandibular advancement: a five-year retrospective study. Int J Oral Maxillofac Surg.

[36] Tan SK, Leung WK, Tang ATH, Zwahlen RA (2017). How does mandibular advancement with or without maxillary procedures affect pharyngeal airways? An overview of systematic reviews. PLoS One.

[37] Conradt R, Hochban W, Brandenburg U, Heitmann J, Peter JH (1997). Long-term follow-up after surgical treatment of obstructive sleep apnoea by maxillomandibular advancement. Eur Respir J.

[38] Li KK, Powell NB, Riley RW, Troell RJ, Guilleminault C (2000). Long-term results of maxillomandibular advancement surgery. Sleep Breath.

[39] Jones R, Badlani J, Jones C (2010). Maxillary, mandibular and chin advancement surgery for the treatment of obstructive sleep apnoea. Aust Dent J.

[40] Dekeister C, Lacassagne L, Tiberge M, Montemayor T, Migueres M, Paoli JR (2006). Mandibular advancement surgery in patients with severe obstructive sleep apnea uncontrolled by continuous positive airway pressure. A retrospective review of 25 patients between 1998 and 2004. Rev Mal Respir.

[41] Jaspers GW, Booij A, de Graaf J, de Lange J (2013). Long-term results of maxillomandibular advancement surgery in patients with obstructive sleep apnoea syndrome. Br J Oral Maxillofac Surg.

[42] Ronchi P, Cinquini V, Ambrosoli A, Caprioglio A (2013). Maxillomandibular advancement in obstructive sleep apnea syndrome patients: a restrospective study on the sagittal cephalometric variables. J Oral Maxillofac Res.

[43] Hsieh YJ, Liao YF, Chen NH, Chen YR (2014). Changes in the calibre of the upper airway and the surrounding structures after maxillomandibular advancement for obstructive sleep apnoea. Br J Oral Maxillofac Surg.

[44] Boyd SB, Walters AS, Waite P, Harding SM, Song Y (2015). Long-term effectiveness and safety of maxillomandibular advancement for treatment of obstructive sleep apnea. J Clin Sleep Med.

[45] Vigneron A, Tamisier R, Orset E, Pepin JL, Bettega G (2017). Maxillomandibular advancement for obstructive sleep apnea syndrome treatment: long-term results. J Craniomaxillofac Surg.

[46] Veys B, Pottel L, Mollemans W, Abeloos J, Swennen G, Neyt N (2017). Three-dimensional volumetric changes in the upper airway after maxillomandibular advancement in obstructive sleep apnoea patients and the impact on quality of life. Int J Oral Maxillofac Surg.

[47] de Ruiter MHT, Apperloo RC, Milstein DMJ, de Lange J (2017). Assessment of obstructive sleep apnoea treatment success or failure after maxillomandibular advancement. Int J Oral Maxillofac Surg.

[48] Eikermann M, Jordan AS, Chamberlin NL, Gautam S, Wellman A, Lo YL, White DP, Malhotra A (2007). The influence of aging on pharyngeal collapsibility during sleep. Chest.

[49] Jura M, Kozak LP (2016). Obesity and related consequences to ageing. Age (Dordr).

[50] Ware JC, McBrayer RH, Scott JA (2000). Influence of sex and age on duration and frequency of sleep apnea events. Sleep.

[51] Pillar G, Malhotra A, Fogel R, Beauregard J, Schnall R, White DP (2000). Airway mechanics and ventilation in response to resistive loading during sleep: influence of gender. Am J Respir Crit Care Med.

[52] Burschtin O, Wang J (2016). Testosterone deficiency and sleep apnea. Urol Clin North Am.

[53] Lozo T, Komnenov D, Badr MS, Mateika JH (2017). Sex differences in sleep disordered breathing in adults. Respir Physiol Neurobiol.

[54] Behan M, Wenninger JM (2008). Sex steroidal hormones and respiratory control. Respir Physiol Neurobiol.

[55] Romero-Corral A, Caples SM, Lopez-Jimenez F, Somers VK (2010). Interactions between obesity and obstructive sleep apnea: implications for treatment. Chest.

[56] Young T, Palta M, Dempsey J, Skatrud J, Weber S, Badr S (1993). The occurrence of sleep-disordered breathing among middle-aged adults. N Engl J Med.

[57] Pahkala R, Seppa J, Ikonen A, Smirnov G, Tuomilehto H (2014). The impact of pharyngeal fat tissue on the pathogenesis of obstructive sleep apnea. Sleep Breath.

[58] Nashi N, Kang S, Barkdull GC, Lucas J, Davidson TM (2007). Lingual fat at autopsy. Laryngoscope.

[59] Kim AM, Keenan BT, Jackson N, Chan EL, Staley B, Poptani H, Torigian DA, Pack AI, Schwab RJ (2014). Tongue fat and its relationship to obstructive sleep apnea. Sleep.

[60] Isono S (2012). Obesity and obstructive sleep apnoea: mechanisms for increased collapsibility of the passive pharyngeal airway. Respirology.

[61] Stuck BA, Maurer JT (2017). Recent developments in the diagnosis and treatment of obstructive sleep apnea : English version. HNO.

